# A Benchmark Dataset for Satellite-Based Estimation and Detection of Rain

**DOI:** 10.1038/s41597-026-06565-0

**Published:** 2026-01-15

**Authors:** Simon Pfreundschuh, Malarvizhi Arulraj, Ali Behrangi, Linda Bogerd, Alan James Peixoto Calheiros, Daniele Casella, Neda Dolatabadi, Clement Guilloteau, Jie Gong, Christian D. Kummerow, Pierre Kirstetter, Gyuwon Lee, Maximilian Maahn, Lisa Milani, Giulia Panegrossi, Rayana Palharini, Veljko Petković, Soorok Ryu, Paolo Sanó, Jackson Tan

**Affiliations:** 1 Department of Atmospheric Science, Colorado State University, Fort Collins, USA; 2 Earth System Science Interdisciplinary Center, University of Maryland, Maryland, USA; 3 Department of Hydrology and Atmospheric Sciences, University of Arizona, Tucson, USA; 4 NASA Goddard Space Flight Center, Greenbelt, USA; 5 Instituto Nacional de Pesquisas Espaciais, State of São Paulo, São Paulo, Brazil; 6 Institute of Atmospheric Sciences and Climate, Italian National Research Council, Roma, Italy; 7 Department of Civil and Environmental Engineering, University of California Irvine, Irvine, USA; 8 School of Meteorology & School of Civil Engineering and Environmental Science, University of Oklahoma, Norman, USA; 9 Department of Atmospheric Sciences, Kyungpook National University, Buk-gu, Daegu, South Korea; 10 Institute for Meteorology, Leipzig University, Leipzig, Germany; 11 Departamento de Prevención de Riegos y Medio Ambiente, Universidad Tecnológica Metropolitana, Santiago, Chile; 12 Cooperative Institute for Satellite Earth System Studies, University of Maryland, College Park, USA; 13 University of Maryland, Baltimore County, College Park, USA

**Keywords:** Hydrology, Natural hazards

## Abstract

Accurately tracking the global distribution of precipitation is essential for both research and operational meteorology. Satellite observations remain the only means of achieving consistent, global precipitation monitoring. While machine learning has long been applied to satellite-based precipitation retrieval, the absence of a standardized benchmark dataset has hindered fair comparisons between methods. To address this, the International Precipitation Working Group has developed SatRain, the first AI benchmark dataset for satellite-based detection and estimation of rain. SatRain integrates multi-sensor satellite observations from the primary platforms used in precipitation remote sensing with high-quality reference precipitation estimates derived from gauge-corrected ground-based radar composites over the conterminous United States. It offers a standardized evaluation protocol and out-of-distribution testing data from Asia and Europe to enable robust and reproducible comparisons across machine learning approaches. In addition to algorithm evaluation, the diversity of sensors and inclusion of time-resolved geostationary observations make SatRain a valuable foundation for developing next-generation AI models to deliver more accurate global precipitation estimates.

## Background & Summary

Precipitation, the deposition of water in liquid or frozen form from the atmosphere onto the Earth’s surface, is essential for sustaining ecosystems and a wide range of human activity. However, extreme events at both ends of the climatological distribution of precipitation, such as droughts or heavy precipitation, can cause substantial damage to societies and human livelihoods. Monitoring the global distribution of precipitation is therefore critical not only for advancing scientific understanding of the processes that shape precipitation patterns and drive extreme events but also economic planning and civil security. Despite its crucial role in many aspects of economic and social life on Earth, precipitation estimation still faces significant challenges in meeting the needs of hydrological and climate research, as well as operational applications. Precipitation is one of the most difficult atmospheric parameters to measure accurately because its estimation from both satellite and ground-based observations is complicated by several factors: its high spatial and temporal variability; its phase (liquid, solid, or mixed); its microphysical compositions (including particle shape, densities, and sizes); and the difficulties involved in converting radiometric measurements into quantitative precipitation estimates^1^.

Owing to its significance for socio-economic activities and its fundamental role within the climate system, numerous techniques have been developed to monitor and quantify it. The three principal approaches are rain gauges, ground-based radar, and satellite observations. Rain gauges yield direct and highly accurate measurements but are largely confined to continental regions and are often irregularly distributed^2^. In addition, because gauges measure precipitation only at a single point, they cannot adequately represent the spatial structure of rainfall systems. Ground-based weather radars yield spatially-continuous estimates at high spatial resolution but their coverage remains geographically limited. In contrast, satellites offer consistent, near-global observations, making them the only means of obtaining continuous and spatially comprehensive estimates of precipitation.

However, the accuracy with which precipitation can be estimated or detected from satellite observations varies significantly with sensor type and observing conditions^3^. Although a small number of precipitation radars have been deployed to measure precipitation from space, their spatial and temporal coverages are severly limited. Therefore, global precipitation monitoring has to rely mostly on passive sensors. Passive microwave (PMW) sensors operating in the 10-89 GHz range can detect emission signals associated with precipitation particles over the ocean. However, over land the emission signal from precipitation is hardly distinguishable from the high and variable emission signal from the surface. While higher microwave frequencies ( >80 GHz) are less sensitive to surface properties, their information content primarily derives from scattering by large rain drops and ice particles^4^, providing a weaker link to the precipitation at the surface. Furthermore, the spatial resolution achievable with PMW sensors is limited, requiring deployment in low-Earth orbits. As a result, even for satellite missions comprising constellations of multiple sensors, such as the Global Precipitation Measurement (GPM) mission^5^, the revisit times can exceed three hours in the tropics.

By contrast, geostationary satellites offer near-continuous temporal coverage over much of the globe, with spatial resolutions on the order of a few kilometers. Their main limitation is that they operate only in the visible (Vis) and infrared (IR) bands, which are primarily sensitive to the cloud tops and therefore provide a less direct link to surface precipitation than passive microwave observations.

**Fig. 1:**
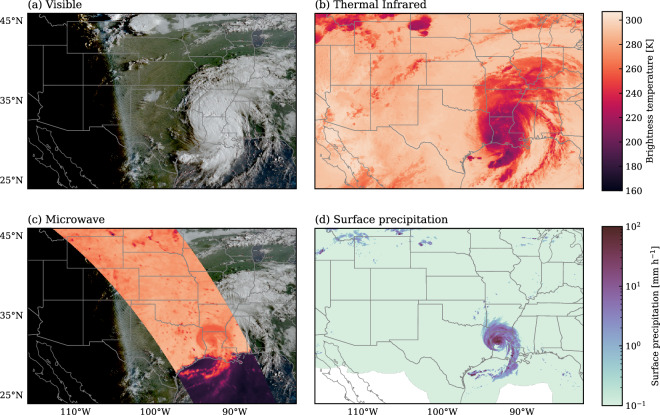
Satellite observations and surface precipitation estimates from the landfall of Hurricane Laura on August 27, 2020, at 12:41 UTC. Panel (a) shows a true-color composite from the Advanced Baseline Imager (ABI) aboard the GOES-16 geostationary satellite. Panel (b) displays geostationary thermal infrared imagery at 11 μm. Panel (c) presents passive microwave observations from the GPM Microwave Imager (GMI) 36.5-GHz, horizontally-polarized channel, overlaid on the GOES true-color RGB. Panel (d) depicts surface precipitation rate estimates from NOAA’s Multi-Radar Multi-Sensor product.

Figure 1 illustrates the key characteristics of the various types of satellite observations used for the remote sensing of precipitation. Panel (a) presents a true-color composite from the Advanced Baseline Imager (ABI^6^) aboard GOES-16, showing Hurricane Laura as an expansive cloud system in the southeastern portion of the domain. Visible imagery such as this true-color composite can reveal detailed cloud structures but is limited to daylight hours due to its reliance on reflected sunlight. Panel (b) displays thermal IR imagery with a wavelength of 11 μm observed from a geostationary platform. At this wavelength, the clear-sky atmosphere exhibits high transmittance, while clouds are opaque. Consequently, the measured radiances primarily originate from the Earth’s surface in cloud-free regions and from cloud tops in cloudy regions. Due to the vertical thermal structure of the atmosphere, the cloud tops appear as areas of cold brightness temperatures against a relatively warmer background. Panel (c) shows horizontally-polarized passive microwave observations at a frequency of 36.5 GHz, corresponding to a wavelength of around 8 mm. Surface-sensitive passive microwave observations are characterized by a strong contrast between ocean and land surfaces. Over the radiatively cold ocean background, Hurricane Laura’s rainband appears as a region of enhanced brightness temperature due to emission from liquid hydrometeors. Over land, the surface itself emits strongly at microwave frequencies, making it difficult to isolate the emission signal from raindrops. Instead, precipitation is primarily detected through the scattering signature of large raindrops and ice particles, which reduce the observed brightness temperatures by attenuating surface emission.

Comparing the three panels to the corresponding radar-based surface precipitation estimates underscores the strength of PMW observations: while Vis and IR imagery primarily depict cloud-top features that may not correlate with surface rainfall, PMW observations exhibit higher spatial coherence with surface precipitation. The principal limitation of PMW imagery, however, is its limited spatiotemporal coverage due to narrower swath widths, longer revisit times, and lower spatial resolution.

### From Satellite Observations to Precipitation Estimates

Satellite observations only contain an indirect signal from the near-surface precipitation and thus require careful processing to produce reliable surface precipitation estimates. This conversion of satellite measurements into precipitation estimates is commonly referred to as retrieval.

While a physics-based formulation of precipitation retrieval algorithms is possible, practical implementations typically require a range of simplifications and ad-hoc assumptions, such as normally distributed retrieval targets and measurement errors or close-to linear relationships between atmospheric variables and satellite observations^7–9^. Because of these difficulties, purely empirical approaches have long been used to directly relate satellite observations to precipitation estimates obtained from other measurement techniques^10–12^. The rise of machine learning (ML) and more recent AI techniques have led to the development of a range of new ML-based algorithms that yield promising results^13–16^.

Satellite-based precipitation retrievals form a core component of widely used merged precipitation products such as the Integrated Multi-satellite Retrievals for GPM (IMERG^17^) and the Global Satellite Mapping of Precipitation (GSMaP^18^). IMERG, for instance, relies on the Goddard Profiling Algorithm (GPROF^8^) to derive precipitation estimates from PMW observations and on the PERSIANN Dynamic Infrared-Rain Rate product^19^ to infer precipitation from geostationary 11 μm IR imagery, merging these inputs into global half-hourly precipitation fields.

### The Need for a Unified Benchmark Dataset

The number of ML-based satellite precipitation retrievals is growing^20,21^, however the algorithms described in the literature are difficult to compare. This is largely because they are typically developed for specific sensors, regions, time periods, and even resolutions. Given the high spatiotemporal variability of precipitation, such differences in sampling and geographic focus significantly influence accuracy metrics, rendering published results incomparable. Moreover, the performance of empirical retrieval algorithms is influenced by the volume, quality, and spatiotemporal sampling of the training and evaluation data, and thus does not solely reflect the intrinsic qualities of a specific algorithm or model.

This lack of comparability makes it difficult to isolate algorithmic improvements driven by advancements in ML from differences introduced by the choice of training and evaluation data. To address this challenge, the International Precipitation Working Group (IPWG), a permanent Working Group of the Coordination Group for Meteorological Satellites (CGMS), has established a ML working group tasked with developing a standardized benchmarking dataset for empirical and ML-based precipitation retrievals^22^. The result of this effort is the Satellite-Based Estimation and Detection of Rain (SatRain) dataset, an AI-ready, large-scale benchmark dataset for the development and evaluation of precipitation retrieval algorithms covering a wide range of observations modalities and multiple climate zones. Despite its name, the dataset is not limited to rain but includes all types of precipitation encountered during the training and testing periods thus providing a comprehensive resource for developing and testing precipitation retrieval algorithms.

### The SatRain Dataset

The SatRain dataset integrates multi-sensor satellite observations with gauge-corrected, ground-based radar precipitation estimates. It provides a large, curated training set over the conterminous United States (CONUS), encompassing diverse climate regimes ranging from subtropical humid regions to arid deserts, mountainous terrain, and temperate to cold continental zones. Data are available both on a 0.036° regular latitude-longitude grid and the native sampling of the passive microwave sensors. All input and reference fields are consistently mapped to these two spatial representations, enabling direct use for both pixel-based and image-based AI algorithms and ensuring the dataset’s AI-readiness. The satellite observations span a wide range of sensing modalities relevant to precipitation remote sensing, including temporally resolved imagery from geostationary platforms. To support model generalization studies, SatRain also includes independent test sets from Korea and Austria, covering distinct climate regimes and incorporating alternative reference measurement techniques.

The SatRain dataset is constructed using the same satellite observations that underpin major global merged precipitation products such as IMERG and GSMaP. Consequently, machine-learning models trained on SatRain can be directly benchmarked against the retrieval algorithms used to generate these products. This alignment creates a clear pathway for transferring methodological advances developed with SatRain into next-generation global precipitation datasets, thereby supporting progress in meteorological research and applications.

## Methods

### Data Sources

The SatRain dataset integrates the following data sources:

- PMW observations,
- Vis and IR observations from geostationary platforms,
- ancillary environmental data,
- reference precipitation estimates from ground-based weather radars over CONUS,
- independent precipitation estimates from ground-based radars over Korea and gauge measurements in Austria.

A key challenge in creating the SatRain dataset was reconciling differences in spatial resolution and sampling across sensors and datasets. To balance flexibility with manageable dataset size, SatRain data is provided in two spatial sampling geometries. The first geometry is a regular latitude-longitude grid at 0.036° resolution. This resolution matches the native grid of a major global geostationary IR dataset^23^ and remains finer than the effective resolution of current satellite precipitation products. The second is the *on-swath* geometry, which retains the native spatial sampling of the PMW base sensor. Because many precipitation retrieval algorithms were originally designed to operate on the native sensor sampling, SatRain retains this format to support these retrieval designs and cross-comparison against existing operational retrievals such as GPROF.

Since PMW observations are only available at discrete overpass times for a given location, SatRain is organized around these overpasses, with coincident data from other sources collocated accordingly. As a result, the available training and testing samples are limited to the overpass times of the underlying PMW sensors.

#### Passive Microwave Observations

The PMW observations used to build the SatRain dataset are sourced from two different sensors: the GPM Microwave Imager (GMI^24^) aboard the GPM Core Observatory, and a selection of channels of the Advanced Technology Microwave Sounder (ATMS^25^) aboard the NOAA-20 satellite^26^. The GMI and ATMS sensors were chosen to represent two ends of the spectrum of PMW instrumentation used for measuring precipitation. GMI, the flagship PMW sensor of the GPM constellation, has been designed for precipitation remote sensing and features optimized spectral coverage and comparably high spatial resolution. In contrast, ATMS was developed primarily for operational weather forecasting and, compared to GMI, offers fewer precipitation-sensitive channels and significantly coarser spatial resolution.

**Fig. 2:**
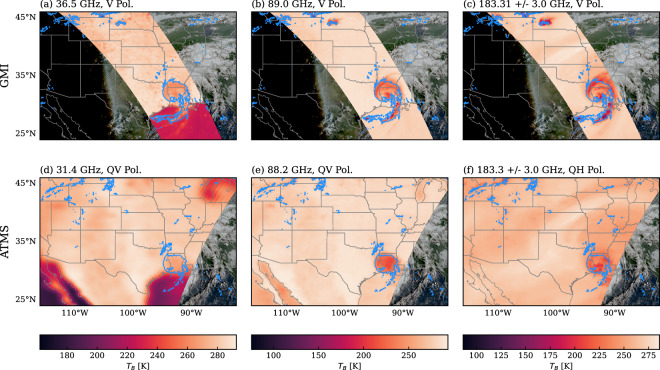
Passive microwave observations from the GMI (**a–c**) and ATMS (**d–f**) sensors from the landfall of Hurricane Laura on August 27, 2020. The top row displays three selected channels from the GMI sensor, highlighting its high-resolution but limited swath. The bottom row presents corresponding channels from the ATMS sensor for comparison. The blue contour line encloses regions with surface precipitation rates exceeding 1 mm h^−1^.

Figure 2 compares GMI and ATMS observations of Hurricane Laura, collected at 12:41 UTC and 08:41 UTC, respectively, on August 27, 2020. GMI offers higher spatial resolution than ATMS, allowing finer cloud and precipitation structures to be resolved, but this advantage comes at the cost of a narrower swath and thus reduced spatial coverage. The instruments also differ in their scanning strategies. GMI operates as a conical scanner, with its antenna beam sweeping out a cone relative to the spacecraft, producing near-circular scans on the surface at a nearly constant Earth-incidence angle. This geometry ensures uniform polarization and viewing characteristics across the swath. ATMS, by contrast, employs a cross-track scanning approach, in which the antenna beam sweeps perpendicular to the spacecraft track. For cross-track sensors, footprint size increases with scan angle, resulting in coarser resolution toward the swath edges, while variations in path length and polarization angle introduce additional systematic differences across the scan.

The PMW observations in SatRain are taken from the calibrated brightness temperature products of the GPM mission for GMI^27^ and from ATMS aboard the NOAA-20 satellite^28^. The channels used in the dataset are summarized in Table 1, along with their polarizations and approximate footprint sizes. Because the GPM data product does not include the ATMS temperature-sounding channels near 50 GHz, these channels are excluded from the SatRain ATMS subset.

**Table 1:** Frequencies (Freq.), polarizations (Pol.), and footprint (FP) sizes of the PMW observations included in the SatRain dataset.

GMI		
Freq. [GHz]	Pol.	FP [km × km]
10.65	V	32 × 19
10.65	H	32 × 19
18.7	V	18 × 11
18.7	H	18 × 11
23.8	V	15 × 9.2
36.5	V	14 × 8.6
36.5	H	14 × 8.6
89.0	V	7.2 × 4.4
89.0	H	7.2 × 4.4
166.5	V	7.2 × 4.4
166.5	H	7.2 × 4.4

ATMS		
Freq. [GHz]	Pol.	FP [km]
23.800	QV	75
31.400	QV	75
88.2	QV	32
165.5	QH	16
183.31 ± 7.0	QH	16
183.31 ± 4.5	QH	16
183.31 ± 3.0	QH	16
183.31 ± 1.8	QH	16
183.31 ± 1.0	QH	16

For GMI, footprint sizes are reported as the full width at half maximum (FWHM) along and across the boresight direction. For ATMS, only the nadir FWHM is given; at nadir the footprint is approximately circular and therefore represented by a single value. The polarization of ATMS are denoted quasi-horizontal (QH) and quasi-vertical (QV) as the polarization mixture changes with the sensor viewing angle.

#### Geostationary Visible and Infrared Observations

Vis and IR sensors benefit from the shorter wavelengths of the radiation they measure, enabling much higher spatial resolution and deployment on geostationary platforms. From this vantage point, they provide near-continuous coverage of the underlying hemisphere. Consequently, geostationary observations serve as a critical complement to PMW observations for real-time and continuous precipitation monitoring.

**Fig. 3:**
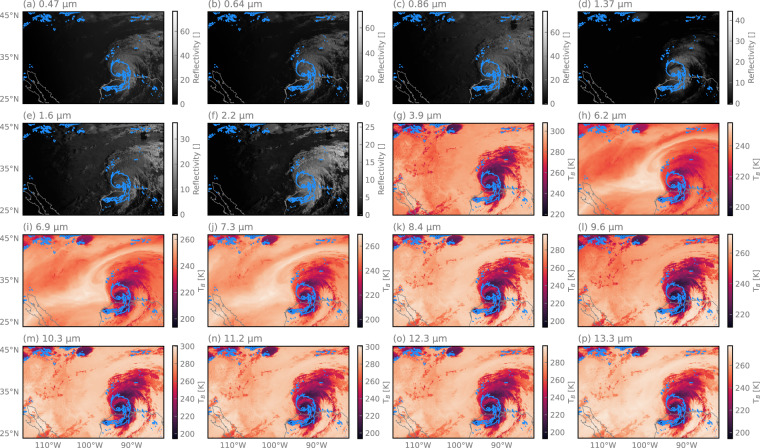
The 16 channels of the GOES Advanced Baseline Imager (ABI) observing Hurricane Laura during landfall on August 27, 2020, at 08:41 UTC. The first six channels primarily measure reflected solar radiation. The remaining channels measure infrared radiation emitted from the atmosphere. Observations from geostationary sensors have the advantage of providing close-to continuous coverage but are sensitive primarily to cloud-top properties and thus only provide limited information on precipitation close to the surface. The blue contour encloses areas where surface precipitation exceeds 1 mm h^−1^.

Figure 3 shows observations from the 16 spectral channels of the ABI sensor aboard GOES-16 during the landfall of Hurricane Laura. The native spatial resolution of ABI channels ranges from 500 meters to 2 kilometers at the sub-satellite point on the equator. Although this resolution degrades over CONUS due to increasing viewing angles, it remains higher than that of PMW sensors. The first six channels are Vis and near-IR bands that measure reflected solar radiation and are only available during daylight hours. The remaining ten channels operate in the thermal IR and provide data continuously, both day and night. Among the thermal IR bands, the main distinguishing feature is their sensitivity to atmospheric water vapor. For instance, channels centered at 6.2, 6.9, and 7.3 μm are more sensitive to upper-tropospheric moisture. This sensitivity to water vapor provides contextual information on the moisture content of the air but reduces the penetration depth of the observations.

A particular challenge related to incorporating geostationary observations into global precipitation retrievals is that the channel availability changes between sensors and thus the geographical coverage regions. SatRain integrates data from CONUS, the Korean peninsula, and Austria, which are covered by different geostationary platforms. While observations over CONUS are derived from the GOES-16^29^ platform, observations over Korea are derived from the Himawari-8 and -9 platforms^30^, and observations over Austria from the Meteosat-10 platform^31^. Table 2 lists the central wavelengths for the channels included from the ABI sensor on GOES-16, the Advanced Himaware Imager (AHI^32^), and the SEVIRI^33^ sensor on Meteosat-10. The observations from the ABI, AHI, and SEVIRI sensors were downloaded from^34–36^, respectively.

**Table 2:** Channel central wavelengths ABI sensor on GOES-16, the AHI sensor on Himawari-8/9, and the SEVIRI sensor on Meteosat-10.

Channel	ABI	AHI	SEVIRI
1	0.47	0.455	0.75
2	0.64	0.51	0.635
3	0.86	0.645	0.81
4	1.38	0.86	1.64
5	1.61	1.61	3.92
6	2.26	2.26	6.25
7	3.9	3.85	7.35
8	6.15	6.25	8.7
9	7.0	6.95	9.66
10	7.4	7.35	10.8
11	8.5	8.6	12.0
12	9.7	9.63	13.4
13	10.3	10.45	
14	11.2	11.20	
15	12.3	12.35	
16	13.3	13.3	

Latest-generation geostationary platforms provide observations at temporal resolution of at least 10 minutes allowing them to closely track the evolution of precipitation systems. In order to allow users to explore the temporal information content in time-resolved geostationary observations, the SatRain dataset includes observations from multiple time steps around the overpass of the PMW sensor.

In addition to the multi-channel Vis and IR observations from the latest generation of geostationary sensors, the SatRain dataset also integrates 0.036° gridded thermal IR observations from the 11 μm infrared window sourced from the Climate Prediction Center (CPC) global gridded geostationary IR dataset^23^. Since these observations are available almost continuously from 1998, they play an important role for generating long-term precipitation records and are included as an independent input data source in the SatRain dataset.

#### Ancillary Data

**Fig. 4:**
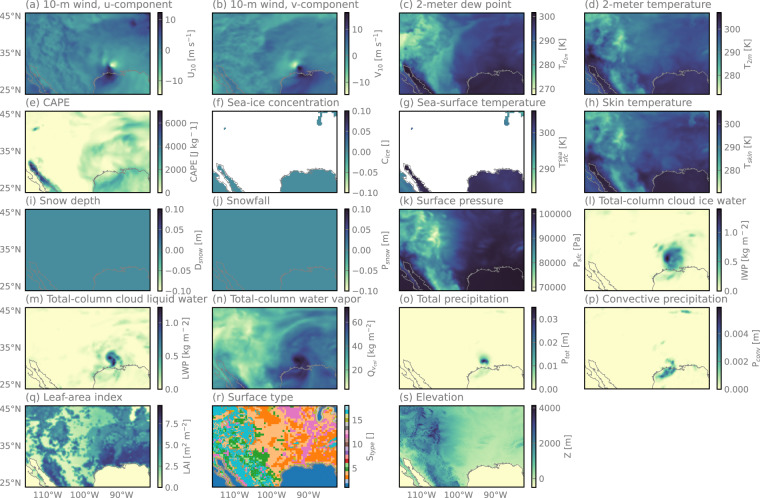
Ancillary variables provided by the SatRain dataset for the scene depicted in Fig. 1. Panels (a) to (q) show the dynamic ERA5 fields included in the ancillary data. Panel (r) shows the 18-class surface classification from the GPROF algorithm and panel (s) the surface elevation from the NOAA Globe dataset.

Because the relationship between satellite observations and surface precipitation is often under-constrained, it is common to augment satellite observations with complementary environmental information, so-called ancillary data, to improve the accuracy of the precipitation estimates. Typical examples include the surface type, atmospheric and surface temperatures, humidity, and elevation. The SatRain dataset includes several static and dynamic ancillary variables. Dynamic ancillary data describing the state of the atmosphere and the surface are derived from the ERA5^37^ dataset. In addition to that, the ancillary data also contains an 18-class surface classification that has been developed for the GPROF precipitation retrieval^38^ combing microwave-based surface-type information with snow- and sea-ice coverage data from the Autosnow product^39^. In terms of static variables, SatRain provides the surface elevation sourced from the NOAA Global Land One-kilometer Base Elevation (GLOBE) digital elevation model^40^. Figure 4 showcases the ancillary variables included in the SatRain dataset for the landfall of Hurricane Laura.

#### Precipitation Reference

**Fig. 5:**
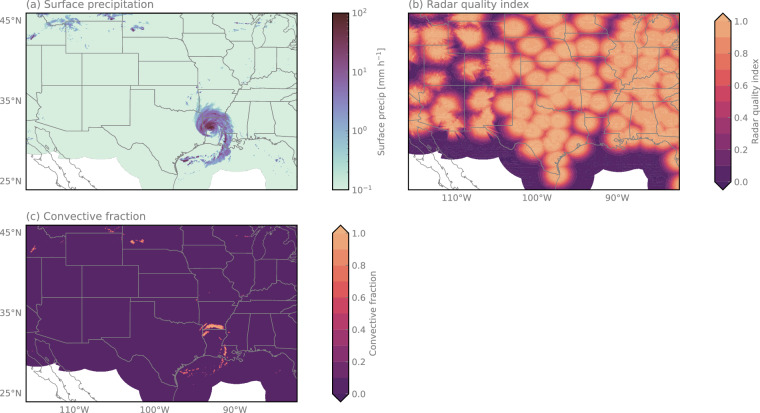
Reference precipitation estimates and auxiliary fields during the landfall of Hurricane Laura. Panel (a) shows surface precipitation estimates from NOAA’s gauge-corrected Multi-Radar Multi-Sensor product. Panel (b) displays the radar quality index quantifying the reliability of the precipitation estimates. Panel (c) presents the convective fraction field, illustrating the hydrometeor classification data included in the SatRain dataset.

The precipitation reference used in the SatRain dataset is derived from gauge-corrected ground-based precipitation radar measurements from NOAAs Multi-Radar Multi-Sensor (MRMS^41^) product. MRMS is based on radar observations from NEXRAD, the most extensive network of precipitation radars in the world comprising around 160 polarimetric S-band radars. The radar-derived estimates of liquid precipitation are corrected using hourly gauge-correction factors thus enforcing consistency between instantaneous estimates and direct measurements of hourly accumulations from gauge stations. While a certain level of residual uncertainty in these estimates cannot be eliminated, they are generally considered to be the best currently available estimates of surface precipitation with near-complete coverage over CONUS. In addition to reference surface precipitation rates, the SatRain dataset contains a radar quality index and the gauge correction factor, allowing the user to customize the quality requirements for the radar estimates used during both training and evaluation. Furthermore, the dataset contains precipitation-type masks identifying convective and stratiform rain, snow, and hail as provided by the MRMS data. Examples of these fields are shown in Fig. 5.

#### Independent Precipitation Reference

The SatRain training data is derived from four years (2018-2021) of ground-based radar measurements over CONUS. While this region encompasses a wide range of climate zones, it does not fully capture the diversity of precipitation systems observed globally. To mitigate this limitation, the testing data includes a full year withheld from the training period, as well as two additional test sets derived from distinct geographical regions and independent measurement systems. These complementary datasets help reduce the risk of overfitting to the weather patterns characteristic of the training domain.

The first geographically independent test dataset consists of gauge-corrected, ground-based radar rainfall estimates over South Korea, generated using radar compositing methods optimized for the Korean domain^42^. The Korea-specific merging technique uses radial basis function interpolation to combine radar and rain gauge observations resulting in a high-resolution rainfall product in both space and time^43^. Although the measurement approach is similar to that used in the MRMS reference data over CONUS, the processing of raw data into precipitation estimates differs. The dataset also represents a distinct climate regime from the training data, offering a valuable test of the precipitation retrieval model’s ability to generalize beyond the conditions typically encountered over CONUS. Studies such as^44^ showed that cloud characteristics affecting both PMW radiometers and IR observations differ substantially between Korea and CONUS and can cause significant biases in algorithms applied to these two regions. The independent testing data from Korea thus provides an opportunity to assess whether retrieval improvements are achieved at the cost of global generalizability.

**Fig. 6:**
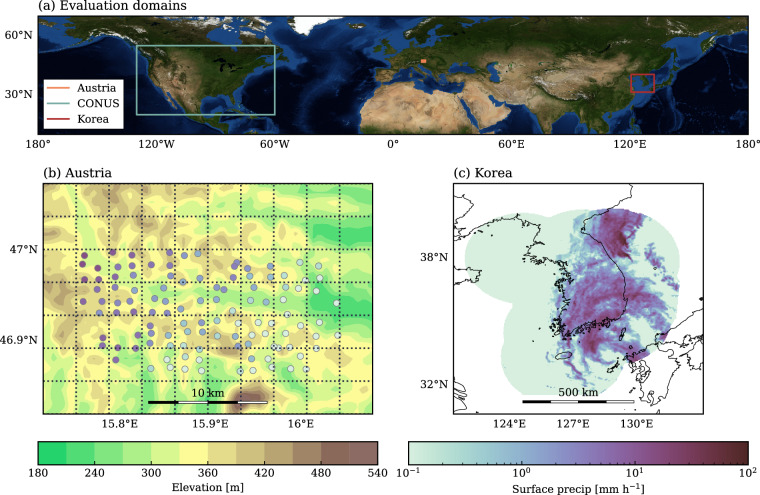
Independent test data included in the SatRain dataset. Panel (a) shows the spatial coverage of the three temporally or spatially independent test datasets. Panel (b) shows the location of the WegenerNet gauge stations used as reference measurements in the Austria domain and the grid they are aggregated to. Panel (c) shows an example in the Korea domain, with radar and rain-gauge data obtained from the Korea Meteorological Administration.

The second independent evaluation dataset is derived from gauge measurements from the WegenerNet^45^ gauge network around the Feldbach region in Austria. While IPWG focuses primarily on gauge-corrected radar data for validation, WegenerNet is unique in that its gauge density is sufficiently high that the addition of radar data would not modify any of the gauge accumulations. In addition, it offers validation over a mountainous regime that radar and gauge networks still struggle with. For the comparison against satellite-based precipitation estimates, the half-hourly accumulations from the ground stations were converted to precipitation rates and aggregated to the 0.036° grid using binning. As can be seen in Panel (b) of Fig. 6, the gauge density is sufficient to cover 24 grid cells with at least two gauges per cell.

### Dataset Generation

The SatRain dataset is generated by extracting observations from all available overpasses of the GMI and ATMS sensors over the target domain and adding the corresponding geostationary observations, ancillary data, and ground-based reference precipitation estimates. The resulting collocation scenes are then used to extract fixed-size training scenes to produce AI-ready training and validation sets suitable for training ML precipitation retrievals.

The SatRain dataset is partitioned into training, validation, and testing subsets that are designed to always be either temporally or spatially independent. Training and validation data are extracted from the years 2018 through 2021, with the first five days of each month allocated to the validation set and the remaining days to the training set. For testing, the CONUS subset uses data from 2022. The independent test set over Austria is based on observations from 2021 and 2022, while the Korea test set covers the period from October 2022 through October 2023.

#### Generation of Collocation Scenes

The first step of the creation of the SatRain dataset consists of the extraction of collocation scenes for every overpass of the PMW base sensor, i.e., GMI or ATMS, over the targeted domain (CONUS, Austria, or Korea). The resulting collocation scene contains all retrieval input data, i.e. satellite observations and ancillary data, combined with the coincident reference precipitation on a shared spatial grid. Two type of collocation scenes are extracted for every overpass of the base sensors: An on-swath scene, which uses the native sampling of the PMW observations, and a gridded scene, which contains all data regridded to a regular latitude-longitude grid with a resolution of 0.036°.

**Fig. 7:**
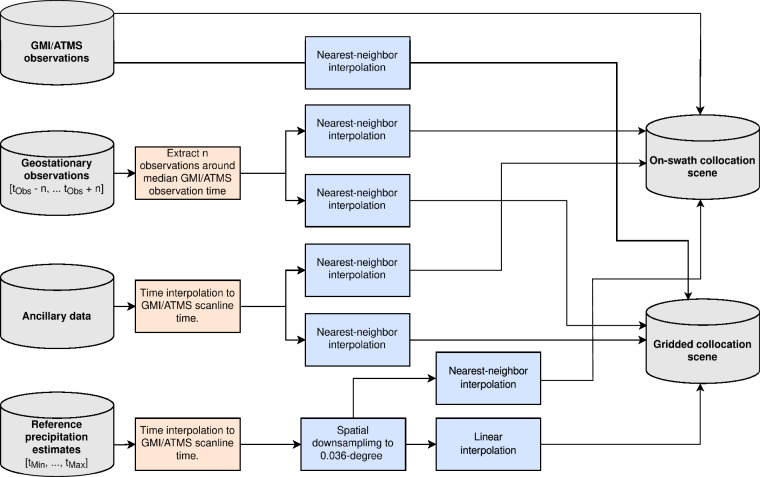
Flow diagram illustrating the data flow for collocating the satellite observations and ground-based reference precipitation estimates for the SatRain dataset.

The collocation process, as illustrated in Fig. 7, starts out with the PMW observation from an overpass of GMI or ATMS over the domain containing the reference data. Corresponding ground-based reference data and ancillary data are extracted to cover the time range of the overpass and interpolated to the observation time of each scan-line of the PMW observations. The MRMS data, which have a native resolution of 0.01°, are reduced in resolution to match the 0.036° grid by smoothing using a Gaussian filter with a full width at half maximum of 0.036° and interpolated linearly to the target grid. The mapping of the reference precipitation estimates to the on-swath geometry is performed by nearest-neighbor interpolation. Similarly, the PMW observations are mapped to the regular latitude-longitude grid by nearest neighbor interpolation.

The global gridded IR geostationary observations are extracted at a temporal resolution of 30 min over a time range of 8 h centered on the median overpass time. Multi-channel Vis and IR observations from the ABI, AHI, and SEVIRI sensors are extracted over a time window of 1 hour and a temporal resolution of 10 minutes. The geostationary observations are mapped to the gridded and on-swath geometries using nearest-neighbor interpolation.

#### Training Scene Extraction

To generate AI-ready training and validation data from the collocation dataset, fixed-size training scenes are extracted from the previously created collocation dataset, separately for the gridded and on-swath subsets. The scenes are extracted randomly, allowing an overlap of up to 50% between neighboring scenes, requiring 75% of the pixels to contain valid observations and reference data. The scene size for the gridded data is 256 pixel  × 256 pixel and 64 pixel  × 64 pixel for the on-swath data. The smaller size of 64 × 64 used for the on-swath data is to accomodate both the GMI sensor, which has 221 pixels per scan, and the ATMS sensor which records only 96 pixel per scan.

**Fig. 8:**
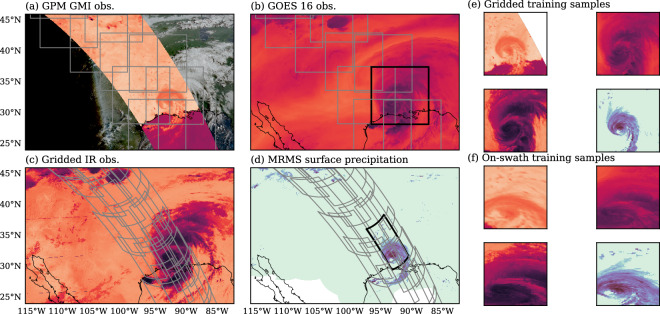
Fixed-size training scenes extracted from the SatRain collocation scenes. Panels (a), (b), and (c) show selected channels from the passive microwave (PMW), visible, and infrared observations that make up the input data of the SatRain dataset. Panel (d) shows the ground-radar-based precipitation reference used as training targets. Grey lines mark the outlines of the training samples extracted from this collocation scene for the gridded and on-swath observations. Black lines mark the sample training scenes displayed in Panel (e) and (f).

Figure 8 shows an example collocation scene from a GMI overpass of Hurricane Laura at 12:41 UTC on August 28, 2020. The grey boxes mark the randomly extracted fixed-size training scenes in both the gridded and on-swath geometries. Due to the irregular sampling pattern of PMW sensors, the on-swath scenes appear distorted when displayed in the equirectangular projection used for the figures. Training patches are restricted to the PMW swath to ensure that each sample includes valid PMW observations. Although this particular example covers only a small portion of the CONUS, the PMW swath shifts with each orbit, and the resulting training, validation, and testing datasets collectively span the full CONUS domain.

## Data Records

### Dataset Organization

The SatRain dataset is organized in a hierarchical folder structure that mirrors its conceptual structure. At the top level, it is divided into two independent subsets corresponding to collocation scenes from GMI overpasses and ATMS observations, with the underlying PMW instrument referred to as the *base sensor*. For each base sensor, the data are partitioned into training, validation, and testing splits in line with machine learning best practices.

To accommodate different computational and storage constraints, the training and validation data are further subdivided into size-based subsets (‘xs’, ‘s’, ‘m’, ‘l’, and ‘xl’). This design supports lightweight experimentation on smaller systems as well as large-scale training on modern deep learning architectures. Files are not repeated across the size-based subsets, instead each of the subsets is meant to include the files in the preceeding, smaller subsets. Users aiming to use the xl subset should thus combine files from all subsets.

**Fig. 9:**
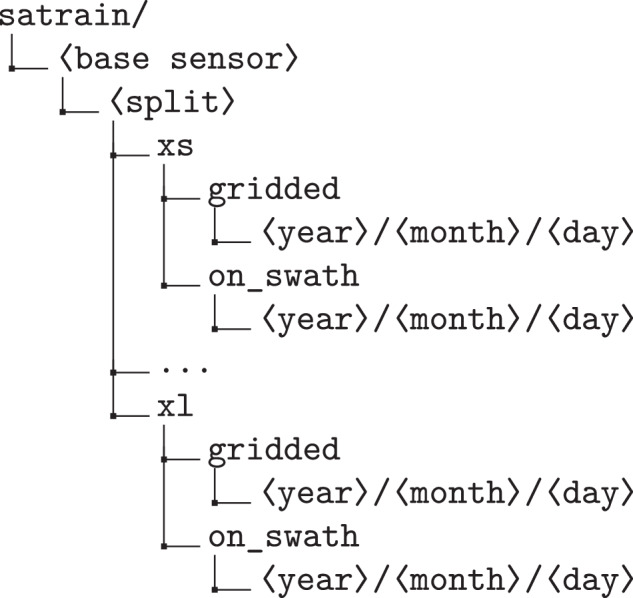
Directory structure of the training and validation splits.

Since all data is available in both gridded and on-swath geometries, the directory is split once more into the data represented using the on-swath and gridded spatial sampling. Finally, files are organized temporally into folders by year, month, and day of the month. The resulting folder hierarchy for the training and validation data is shown in Fig. 9.

**Fig. 10:**
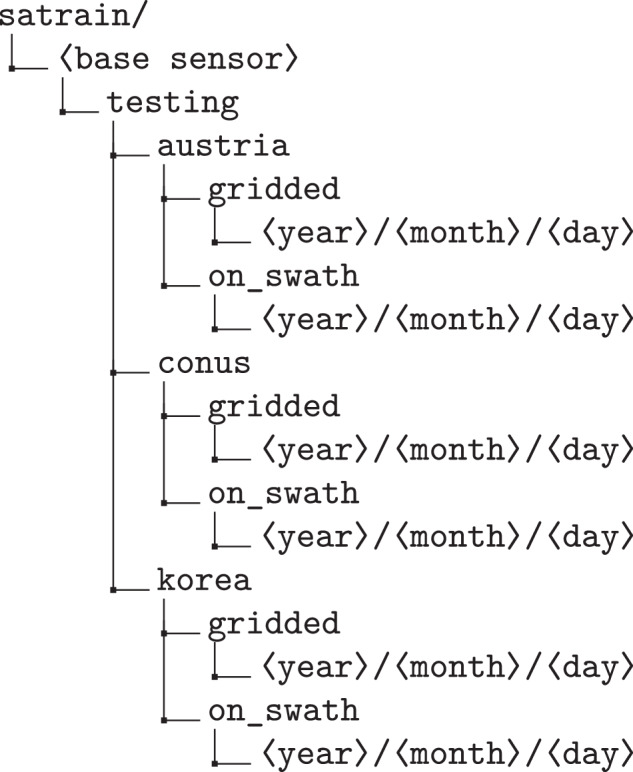
Directory structure of the testing split.

The testing data are organized slightly differently from the training and validation splits. The testing set is not subdivided into size-based subsets, since all evaluations must be performed on the same data to ensure comparability. Instead, the testing data are grouped by the underlying spatial domain: CONUS, Korea, and Austria, which correspond to the available reference data sources: MRMS over CONUS, ground-based radar data over Korea, and in-situ measurements from the WegenerNet stations in Austria. The resulting folder hierarchy is illustrated in Fig. 10.

A further distinction between the training and validation data and the testing data is that the collocation scenes in the testing set are not divided into fixed-size patches. The testing data preserves the original observation structure to avoid sampling distortions, simplify comparisons between gridded and on-swath retrievals, and enable direct evaluation of existing precipitation products on the test data.

### Data Files

The various input and target data for each training scene are stored in separate NetCDF4 files. This modular organization allows users to download just the data they intend to use, for example, only the small (‘s’) subset of the GMI and target data to train and evaluate a retrieval using only PMW observations. Each file is identified using an individual prefix (‘gmi_’, ‘atms_’, ‘geo_’, ‘geo_ir_’, ‘ancillary_’,‘target_’) following a time stamp in the format YYYYMMDDHHMMSS containing the median observation time. Input and target files corresponding to a specific training, validation, or testing sample can thus be identified using this timestamp.

Due to the large size of the time-resolved geostationary observations, the geostationary observations are split into files containing only the observations closest to the reference precipitation estimates and files containing observations from multiple observations times. The multi-timestep observations are stored in separate files with the suffix ‘_t’, i.e., ‘geo_t_< timestamp>.nc’ and ‘geo_ir_t_< timestamp>.nc’.

#### PMW Observations

The PMW observations in the SatRain dataset consist of observations from the GMI sensor and the ATMS sensor on the NOAA-20 satellite. They are stored in files labeled ‘gmi_< timestamp>.nc’ for the subset using GMI as base sensor and, correspondingly, ‘atms_<timestamp>.nc’ for the subset using ATMS as base sensor. Each file contains the brightness temperatures in Kelvin for each channel (observations), the corresponding earth-incidence angles in degree (earth_incidence_angle), and the observation time (scan_time) corresponding to each scan line. The channels included for the GMI and ATMS sensors are listed in Table 1.

### Geostationary Vis and IR Observations

The multi-channel, geostationary Vis and IR observations from the GOES, Himawari, and Meteosat platforms are stored in files named ‘geo_< timestamp>.nc’. These files contain the observations closest in time to the measurement time of the reference precipitation estimates in the variable observations. The visible channels are stored as reflectances while thermal IR channels are stored using brightness temperatures. The geostationary data currently do not include viewing angles so the user will have to add them manually. The multi-timestep files (geo_t_< timestamp >.nc’) contain observations from four 10-minute time steps prior to the collocation median time and three 10-minute time steps after the collocation median time.

Since GOES observations are only available over CONUS, the geostationary observations for the test data from the Korea and Austria domains are derived from the AHI sensor onboard Himawari-8 and -9 and the SEVIRI sensor onboard Meteosat-10. Table 2 lists the central wavelengths of the channels of each of the included sensors. Since the sensors have different channels, the observations differ in their spectral coverage and users will need to account for that in their algorithm design.

### Geostationary IR Observations

The single-channel gridded IR observations from the global, merged CPC dataset^23^ are stored in files named ‘geo_ir_< timestamp>.nc’ and contain IR window-channel observations from wavelengths around 11 μm. The observed brightness temperatures in K are stored in the variable observations. The single-timestep files contain the Geo IR observations closest to the measurement time of the reference precipitation estimates. The temporally-resolved Geo IR observations contain 16 half-hourly observations centered on the median observation time and are stored in files named ‘geo_ir_t_< timestamp>.nc’.

### Ancillary Data

The ancillary data is stored in separate files named ‘ancillary_< timestamp >.nc’. Each file contains the ancillary variables listed in Table 3.

**Table 3:** List of the variables, their explanations, units, and data sources included in the ancillary data.

Variable name	Explanation	Unit	Source
ten_meter_wind_u	Zonal wind at 10 m altitude	m s^−1^	ERA5
ten_meter_wind_v	Meridional wind at 10 m altitude	m s^−1^	ERA5
two_meter_dew_point	Dew-point temperature at 2 m altitude	K	ERA5
two_meter_temperature	Near-surface temperature	K	ERA5
cape	Convective available potential energy	J kg^−1^	ERA5
sea_ice_concentration	Fractional sea-ice coverage	—	ERA5
sea_surface_temperature	Sea surface temperature	K	ERA5
skin_temperature	Surface skin temperature	K	ERA5
snow_depth	Snow depth	kg m^−2^	ERA5
snow_fall	Snowfall rate	m h^−1^	ERA5
surface_pressure	Surface pressure	hPa	ERA5
total_column_cloud_ice_water	Vertically integrated mass of cloud ice	kg m^−1^	ERA5
total_column_cloud_liquid_water	Vertically integrated mass of liquid cloud droplets	kg m^−2^	ERA5
total_column_water_vapor	Vertically integrated mass of water vapor	kg m^−2^	ERA5
total_precipitation	Total precipitation	m h^−1^	ERA5
convective_precipitation	Convective precipitation	m h^−1^	ERA5
leaf_area_index	Half of the total green leaf area per unit ground area	m^2^ m^−2^	ERA5
surface_type	GPROF 18-class dynamic surface classification	—	GPROF V7
elevation	Surface elevation	m	NOAA GLOBE

### Reference Data

The reference data for every scene are stored in a file ‘target_< timestamp>.nc’, where the timestamp matches that of the input data. The reference data files contain the surface precipitation in a variable called ‘surface_precip’. Additionally, the reference data files derived over CONUS contain several quality indicator variables that can be used to filter the reference data samples used for training and validation. The primary quality indicator is the radar quality index (radar_quality_index), which provides an estimate of the quality of the surface precipitation estimates based on the radar beam height, beam blockage, and the height of the freezing level. Moreover, the files contain the gauge correction factor (gauge_correction_factor) that was applied to correct the radar-only precipitation estimates. Finally, the files also contain the fraction of valid (valid_fraction), snowing (snow_fraction), and hailing (hail_fraction) 0.01°-resolution pixels within the downsampled 0.036° grid box of the SatRain dataset. Since the radar data over Korea and the station data over Austria do not provide this additional information, these auxiliary fields are not provided by the target data files from the Korea and Austria domains.

## Technical Validation

To assess the technical consistency of the dataset, we trained three precipitation retrieval models on the CONUS-based training data, each relying on one of the primary input observation types: single-channel IR data from the CPCIR dataset (Geo-IR), multi-channel Vis and IR geostationary observations from the GOES ABI (Geo), and PMW observations from GMI (GMI). All retrievals rely solely on satellite observations and exclude the ancillary data included in SatRain. Each retrieval is implemented U-Net-type encoder-decoder based on the EfficientNet-V2 architecture^46^ with 14 million parameters.

The SatRain-based ML retrievals are evaluated against two baseline precipitation datasets. The first is the version 7 of the GPROF retrieval (GPROF V7), the current operational PMW algorithm for the GPM constellation that underpins the precipitation estimates used in the IMERG gridded product. The second baseline is the ERA5 reanalysis, which offers continuous global precipitation fields and therefore serves as a natural reference for assessing satellite-based precipitation algorithms.

### Case Studies

We first demonstrate retrieval results for two case studies from the test data of the SatRain dataset: A convective squall line over western CONUS and the landfall of Typhoon Khanun over Korea.

**Fig. 11:**
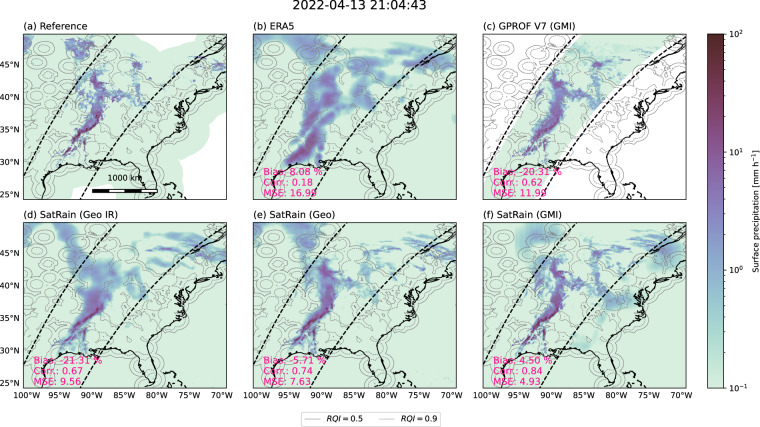
Precipitation retrievals for a quasi-linear convective system, comparing models trained on the SatRain dataset with ground-based precipitation estimates and two baseline products. Panel (a) shows the ground-based reference precipitation, panel (b) the ERA5 reanalysis, and panel (c) the GPROF V7 retrieval applied to GMI observations. Panels (d), (e), and (f) present SatRain-trained retrievals using single-channel IR observations (Geo-IR), multi-channel Vis and IR observations (Geo), and GMI PMW observations, respectively.

Figure 11 presents the reference and retrieved surface precipitation fields for a quasi-linear convective system that developed on the afternoon of April 13, 2022 over the Southern and Midwestern United States. The reference field shows scattered pockets of intense convection in the southern portion of the domain, which merge into a quasi-linear system farther north. In contrast, weaker, scattered patches of stratiform precipitation occur in the northernmost part of the scene.

Both the GPROF V7 baseline estimates and the SatRain-based retrievals capture the intense convective precipitation with high fidelity. Larger differences appear in the stratiform region to the north, particularly for the SatRain retrievals relying exclusively on visible and infrared observations. Despite this, all SatRain-based retrievals achieve strong accuracy metrics, with even the single-channel IR model surpassing the GPROF V7 baseline. ERA5 performs least well in representing the convective event: although it produces comparatively high rain rates in the south, these features are displaced relative to the reference, resulting in an elevated mean-squared error and a low linear correlation coefficient.

**Fig. 12:**
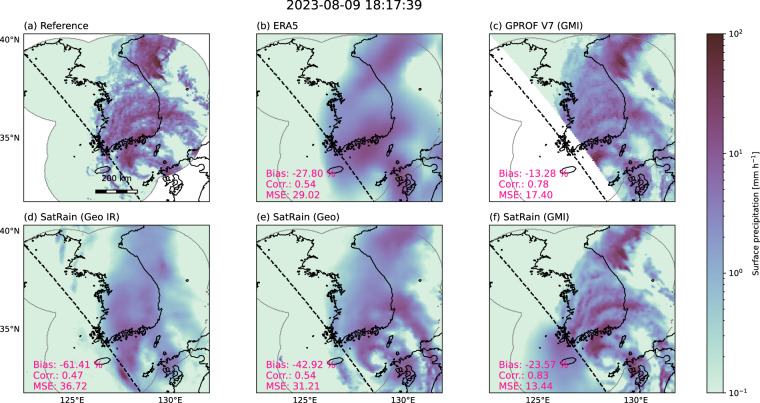
Same layout as Figure 11, but for a case depicting the landfall of Typhoon Khanun over the Korean Peninsula on August 9, 2023, taken from the Korea test-domain dataset.

Figure 12 compares the same retrievals on a test scene from the Korea domain depicting the landfall of Typhoon Khanun on August 9, 2023. All estimates capture the large-scale precipitation features of the Typhoon but with clear differences in accuracy reflecting the information content of their input observations^47^. The PMW-based retrieval performs best, reproducing much of the fine-scale structure evident in the reference estimates. The multi-channel geostationary retrieval captures the primary precipitation bands but misses finer details resolved by the PMW retrieval. In contrast, the single-channel IR retrieval shows the weakest performance, with limited structural detail and correspondingly lower linear correlation and higher mean-squared error.

Compared to the conventional baselines, the SatRain GMI retrieval shows visibly better agreement with the reference precipitation fields than GPROF V7, a result that is supported by scene-based accuracy metrics. The SatRain Vis and IR retrieval achieves accuracy comparable to ERA5 but worse than GPROF V7, which is expected since GPROF V7 leverages passive microwave observations that provide a more direct link to precipitation. The SatRain Geo-IR retrieval performs worse than both baseline methods for this specific case. However, this is consistent with the very limited information content available from its input observations.

### Test-Set Accuracy

**Fig. 13:**
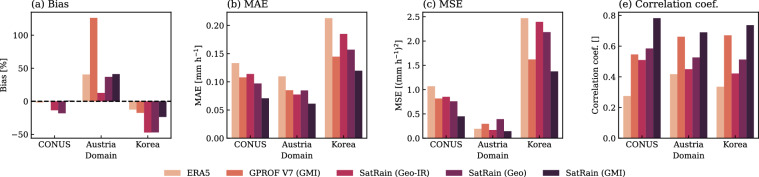
Evaluation of quantitative precipitation estimates from two state-of-the-art precipitation datasets (ERA5 and GPROF) and a U-Net retrieval trained using the different satellite observations of the SatRain dataset. Each retrieval is evaluated for each of the three testing domains of the SatRain dataset. Panels (a) to (d) show the Bias, mean-absolute error (MAE), mean-squared error (MSE), and linear correlation coefficient, respectively.

The ML models trained on the CONUS dataset were further evaluated using all test scenes from the three test regions (CONUS, Austria, and Korea). The test set includes 619 overpasses over the Austria domain, 600 over the Korea domain, and 2912 over the CONUS domain, covering one year for CONUS and Korea and two years for Austria. As shown in Fig. 13, the results align with the case studies discussed above. The SatRain-based GMI retrieval consistently outperforms the GPROF V7 baseline, even though both rely on the same input observations. Retrievals using geostationary measurements (Geo and Geo-IR) exhibit lower overall accuracy but still perform better than the ERA5 baseline.

While the SatRain retrievals demonstrate notable biases over Austria and Korea, these are likely attributable to the exclusive use of CONUS data for training. Such systematic errors align with previous studies documenting the sensitivity of precipitation retrievals to regional precipitation characteristics^44^. Importantly, these biases are not unique to SatRain but also affect operational algorithms such as GPROF V7. Beyond these biases, the relative performance of SatRain versus the baseline retrievals mirrors the results obtained over CONUS. Despite differences in absolute error magnitudes across domains, the ranking of retrieval methods remains largely consistent, suggesting that SatRain-based evaluations generalize well across independent regions, sensors, and time periods.

One exception occurs for the Geo retrieval over Austria, where MSE and MAE values increase relative to other domains. This degradation likely reflects differences between the SEVIRI channels available over Austria and the ABI channels on which the model was trained. Although training was limited to channels with the closest overlap and quantile remapping was applied to harmonize the distributions, discrepancies in channel characteristics and calibration still reduced performance. Accommodating such inter-sensor differences remains an open challenge for machine learning-based precipitation retrievals, and the SatRain dataset offers a platform to investigate these issues further.

**Fig. 14:**
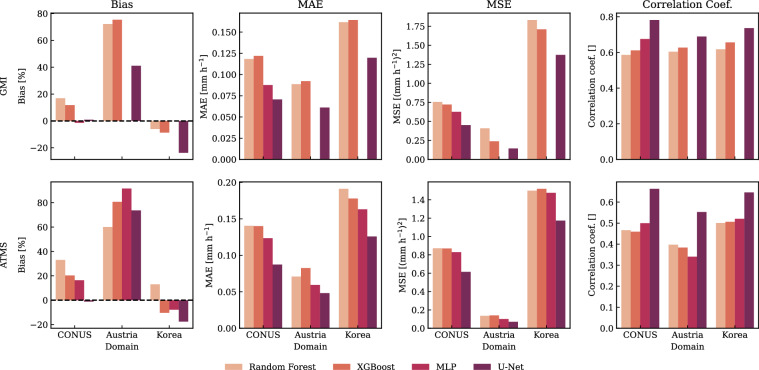
Like Fig. 13 but for four different machine-learning techniques trained on GMI (first row) and ATMS (second row) observations.

In addition to cross-sensor comparisons, we also used SatRain to evaluate the performance of different machine learning approaches. Using GMI and ATMS observations, we trained four retrieval models based on Random Forests, XGBoost, a multi-layer perceptron (MLP), and the U-Net architecture assessed above. Their performance, shown in Fig. 14, reveals a consistent ranking across both sensors and all three domains with the U-Net-based models clearly outperforming the other techniques. Although the relative ordering of Random Forests, XGBoost, and MLP varies by region, their performance differences remain modest. These findings underscore SatRain’s utility as a robust and reliable benchmark for assessing the skill of machine learning-based precipitation retrievals.

We also evaluated the ability of the SatRain-based U-Net retrievals to detect precipitation and heavy precipitation using thresholds of 0.1 mm h^−1^ and 10 mm h^−1^, respectively. For the assessment, we distinguish between probabilistic detection (predicted using probabilities) and deterministic detection (predicted using binary classifications). To enable the SatRain U-Net models to produce probabilistic detection outputs, they were trained with a quantile loss^48^. The predicted quantiles were then used to compute exceedance probabilities for the precipitation and heavy precipitation thresholds. Deterministic classifications were obtained by applying a 50% probability threshold.

**Fig. 15:**
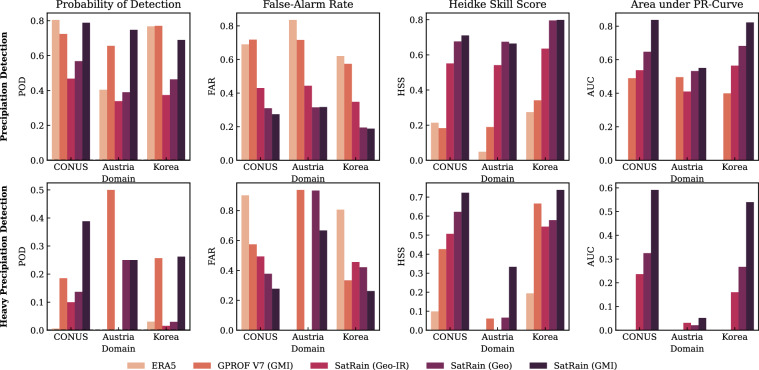
Detection skill for precipitation (first row) and heavy precipitation (second row) for the ERA5 and GPROF V7 baselines and a GMI-based U-Net retrieval trained on the SatRain data. Columns 1 to 3 show the probability of detection (POD), false-alarm rate (FAR), and Heidke Skill Score (HSS) for the deterministic detection. Column 4 shows the area under the precision-recall curve used to assess the probabilistic detection skill.

Figure 15 summarizes the detection performance of the various retrievals. ERA5 total precipitation was converted into binary detection masks by marking areas exceeding the respective threshold as positive. Because ERA5 does not provide probabilistic precipitation information, its probabilistic detection skill cannot be evaluated. GPROF V7, in contrast, provides both precipitation probabilities and a binary precipitation flag, which we use to assess its deterministic and probabilistic detection skill for precipitation detection. Since GPROF V7 does not include a dedicated output for heavy-precipitation detection, heavy-precipitation detections were inferred from regions where the estimated surface precipitation exceeds 10 mm h^−1^.

The resulting detection scores generally mirror the ranking observed for precipitation estimation: SatRain-based models outperform the baselines across most metrics and domains. An exception is the probability of detection (POD), for which ERA5 and GPROF V7 achieve higher values over the CONUS and Korea domains. However, POD is inherently biased because it ignores false alarms. The Heidke Skill Score, which accounts for both false positives and false negatives, provides a more balanced measure of detection performance and confirms the advantage of the SatRain models. Similar patterns are observed for heavy-precipitation detection. All models exhibit low skill over the Austria domain due to the rarity of heavy-precipitation events there. The 619 test scenes contain only four such cases across the two-year testing period. This underscores the challenge of detecting heavy precipitation across climatologically diverse regions.

These results show that the SatRain dataset can be used to train precipitation retrievals that outperform conventional baselines across target domains and tasks and thus demsonstrates the technical consistency of the dataset. The SatRain dataset offers a well-defined framework for training and evaluation thus enabling direct comparison of different ML techniques. The independent test data provide a crucial check, ensuring that performance gains generalize beyond the training domain rather than reflecting overfitting to regional measurement errors or precipitation characteristics. In addition, the ability to evaluate models across sensor types and to combine observations from multiple sensors and time periods makes SatRain a flexible foundation for developing advanced ML-based retrieval methods.

## Usage Notes

**Fig. 16:**
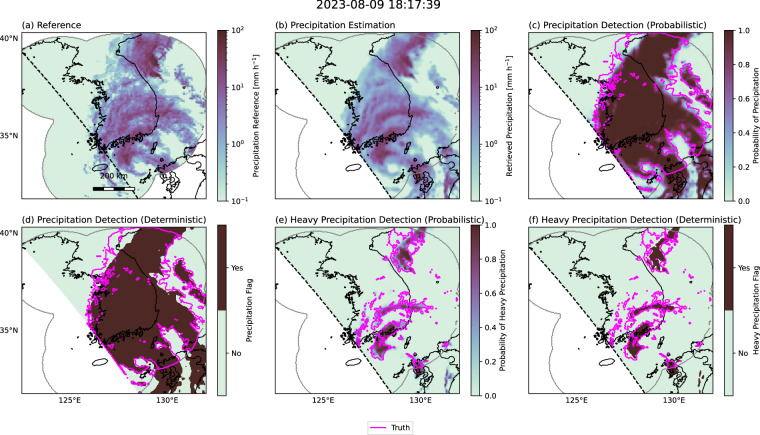
Example retrieval results for the five suggested precipitation estimation and detection tasks during landfall of Typhoon Khanun on August 9, 2023. Panel (b) shows quantitative precipitation estimates retrieved from GMI observations. Panel (c) shows the probability of precipitation for the probabilistic precipitation detection task. Panel (d) shows the precipitation flag for the deterministic precipitation detection task. Panel (e) and (f) show the corresponding probabilistic and deterministic results for the detection of heavy precipitation, defined as precipitation exceeding 10 mm h^−1^.

Different scientific and societal applications may require precipitation retrievals to emphasize specific characteristics, such as retrievals targeting extreme precipitation or specific precipitation types such as snow or hail. While no single benchmark can serve every possible use case, the SatRain dataset defines five distinct evaluation tasks designed to test the ability of ML algorithms to reproduce key aspects of precipitation events. These tasks include: (1) precipitation rate estimation, (2) probabilistic detection of precipitation, (3) deterministic detection of precipitation, (4) probabilistic detection of heavy precipitation, and (5) deterministic detection of heavy precipitation. We adopt thresholds of 0.1 mm h^−1^ and 10 mm h^−1^ to define precipitation and heavy precipitation, respectively. Figure 16 illustrates example results for each of these tasks using the GMI retrieval presented in the previous section.

### Evaluation Protocol

To ensure fair comparison of precipitation retrievals trained on the SatRain dataset, it is essential that models are evaluated using a consistent set of criteria. We therefore propose a standardized evaluation protocol for benchmarking ML retrievals on the SatRain dataset. Users may choose to evaluate their models on all or a subset of the defined tasks. The recommended accuracy metrics for each task are listed in Table 4.

**Table 4:** Precipitation estimation and detection tasks and metrics for evaluating precipitation retrieval methods on the SatRain dataset.

Task	Metrics
Precipitation quantification	Relative bias, mean absolute error, mean squared error, symmetric mean absolute percentage error, linear correlation coefficient, effective resolution
Precipitation detection	Probability of detection, false alarm rate, Heidke Skill Score
Probabilistic precipitation detection	Precision–recall curve
Heavy precipitation detection	Probability of detection, false alarm rate, Heidke Skill Score
Probabilistic heavy precipitation detection	Precision–recall curve

To ensure comparability of benchmark results across algorithms, evaluations should be performed against the gridded reference data. This approach avoids distortions in the evaluation statistics caused by the irregular spatial sampling of on-swath observations. For cross-track scanners, for example, both sampling density and spatial resolution decrease toward the swath edges. The reduced sampling at the swath edges would thus underestimate the effect of the reduced resolution compared to evaluation against the gridded data. To facilitate reproducible comparisons, the gridded reference data files include the scan and pixel coordinates of the nearest swath pixel of the corresponding on-swath file. Using this information, the on-swath retrieval results can be mapped to the gridded reference data easily and consistently.

A reference implementation of the proposed protocol is available through the satrain package. By default, the evaluation compares all retrieval outputs against the reference precipitation estimates on the 0.036° regular latitude-longitude grid, ensuring results are independent of the retrieval’s native coordinate system. For the CONUS domain, evaluations should be restricted to regions with a radar-quality index of at least 0.5 and should include all precipitation types. For the other two domains all reference estimates should be used for the evaluation.

### The satrain package

To facilitate community access and encourage widespread adoption, we developed the satrain Python package. This package streamlines dataset download and management, allowing users to begin working with SatRain without the burden of manual data handling. Comprehensive documentation, including installation instructions, usage examples, and tutorials, is available at satrain.readthedocs.org.

In addition to data access, the package implements SatRain’s standardized evaluation protocol. This enables users to benchmark models trained directly on SatRain and to evaluate independently developed retrievals using the same criteria. A well-defined interface allows all users to leverage the package to automatically conduct evaluations across all testing domains, tasks, and metrics. The satrain package also provides automated tiling functionality to ease the transition from the fixed-size scenes of the training and validation data to the variable-size overpasses used for the testing data.

### Limitations

The SatRain dataset is constructed from high-quality input datasets using state-of-the-art techniques designed to reduce uncertainties in both the satellite observations and the precipitation reference. Despite these efforts, residual uncertainties and measurement errors remain in both components.

On the input side, clearly corrupted satellite imagery is flagged and removed from the satellite-observation data used to construct the SatRain dataset. However, more subtle issues such as undetected artifacts or gradual changes in sensor characteristics may persist and affect the data. These represent practical challenges that any precipitation retrieval must contend with warranting their inclusion in the SatRain dataset.

The precipitation reference data are also subject to non-negligible uncertainties. Although gauge-corrected, ground-based radar composites are widely considered the most reliable spatially continuous precipitation estimates currently available, they are not without error. Beam overshooting, uncertainties in microphysical assumptions, and the inherent difficulty of quantifying snowfall all introduce systematic biases. Snowfall is particularly problematic: most gauges measure it poorly, and MRMS does not apply gauge corrections to snowfall estimates. As a result, snowfall present in the SatRain training and test data should be treated as highly uncertain. For the testing data from the Austria domain, which is derived from the WegenerNet gauge network, only data from heated gauges are used during snowfall, but this reduces gauge density, limits statistical robustness, and does not fully resolve the underlying uncertainties in gauge measurements.

Over ocean, the radar-based reference data are not gauge-corrected, further increasing their uncertainty. In addition, radar beam height increases with distance offshore, making beam overshooting more likely and reducing the reliability of the estimates the farther they are from the coastline.

Further limitations apply to mountain regions. While the SatRain training data covers all of CONUS, including the western mountains, the availability and quality of reference data in these areas are generally lower than in the more densely populated eastern United States. Reduced radar coverage, orographic enhancement, and the occurrence of mixed-phase precipitation processes all contribute additional uncertainty to the reference fields. These limitations inevitably propagate into any machine-learning retrievals developed using the SatRain dataset.

To reduce the impact of these uncertainties on retrieval evaluation, SatRain includes independent test datasets from geographically distinct domains that rely on entirely independent measurement systems. Performance gains that transfer to these independent datasets are more likely to reflect genuine improvements in retrieval capability, rather than overfitting to the same reference data used in training.

It is also important to note that the primary purpose of SatRain is to serve as a benchmark for evaluating and comparing retrieval algorithms, rather than as a basis for developing globally accurate precipitation retrievals. The SatRain dataset is limited to training data over the CONUS and is therefore not designed for the development of global precipitation retrievals. Algorithms trained on SatRain will learn to capture regional precipitation characteristics specific to North America, which may result in substantial biases or retrieval errors when applied to other parts of the world.

## Data Availability

SatRain is available for manual download from rain.atmos.colostate.edu/ipwgml. Additionally, automated download functionality is provided by the satrain^49^ Python package.

## Code availability

The speed package^50^ has been used to create the collocation scenes and extract the training, validation, and testing data for the SatRain dataset. The code to access the data and evaluate retrieval results is available through the satrain package^49^

## References

[1] Levizzani, V. *et al*. Satellite precipitation measurement (Springer, 2020).

[2] Kidd, C. *et al*. So, how much of the earth’s surface is covered by rain gauges? *Bulletin of the American Meteorological Society* **98**, 69–78 (2017). 30008481 10.1175/BAMS-D-14-00283.1 PMC6039978

[3] Stephens, G. L. & Kummerow, C. D. The remote sensing of clouds and precipitation from space: A review. *Journal of the Atmospheric Sciences* **64**, 3742–3765 (2007).

[4] Bennartz, R. & Bauer, P. Sensitivity of microwave radiances at 85–183 ghz to precipitating ice particles. *Radio Science* **38**, 40–1 (2003).

[5] Hou, A. Y. *et al*. The global precipitation measurement mission. *Bulletin of the American Meteorological Society* **95**, 701–722 (2014).

[6] Schmit, T. J. *et al*. Introducing the next-generation advanced baseline imager on goes-r. *Bulletin of the American Meteorological Society* **86**, 1079–1096 (2005).

[7] Boukabara, S.-A. *et al*. Mirs: An all-weather 1dvar satellite data assimilation and retrieval system. *IEEE Transactions on Geoscience and Remote Sensing* **49**, 3249–3272 (2011).

[8] Kummerow, C. D. *et al*. The evolution of the goddard profiling algorithm to a fully parametric scheme. *Journal of Atmospheric and Oceanic Technology* **32**, 2265–2280 (2015).

[9] Maahn, M. *et al*. Optimal estimation retrievals and their uncertainties: What every atmospheric scientist should know. *Bulletin of the American Meteorological Society* **101**, E1512–E1523 (2020).

[10] Griffith, C. G. *et al*. Rain estimation from geosynchronous satellite imagery-visible and infrared studies. *Monthly Weather Review* **106**, 1153–1171 (1978).

[11] Adler, R. F. & Negri, A. J. A satellite infrared technique to estimate tropical convective and stratiform rainfall. *Journal of Applied Meteorology and Climatology* **27**, 30–51 (1988).

[12] Hong, Y., Hsu, K.-L., Sorooshian, S. & Gao, X. Precipitation estimation from remotely sensed imagery using an artificial neural network cloud classification system. *Journal of Applied Meteorology* **43**, 1834–1853 (2004).

[13] Sadeghi, M. *et al*. Persiann-cnn: Precipitation estimation from remotely sensed information using artificial neural networks–convolutional neural networks. *Journal of Hydrometeorology* **20**, 2273–2289 (2019).

[14] Pfreundschuh, S., Ingemarsson, I., Eriksson, P., Vila, D. A. & Calheiros, A. J. P. An improved near-real-time precipitation retrieval for brazil. *Atmospheric Measurement Techniques* **15**, 6907–6933 (2022).

[15] Pfreundschuh, S., Brown, P. J., Kummerow, C. D., Eriksson, P. & Norrestad, T. Gprof-nn: A neural-network-based implementation of the goddard profiling algorithm. *Atmospheric Measurement Techniques* **15**, 5033–5060 (2022).

[16] Afzali Gorooh, V. *et al*. Integrating leo and geo observations: Toward optimal summertime satellite precipitation retrieval. *Journal of Hydrometeorology* **24**, 1939–1954 (2023).

[17] Huffman, G. J. *et al*. Integrated multi-satellite retrievals for the global precipitation measurement (gpm) mission (imerg). In *Satellite precipitation measurement:* Volume 1, 343–353 (Springer, 2020).

[18] Kubota, T. *et al*. Global satellite mapping of precipitation (gsmap) products in the gpm era. In *Satellite precipitation measurement:* Volume 1, 355–373 (Springer, 2020).

[19] Nguyen, P. *et al*. Persiann dynamic infrared-rain rate (pdir-now): A near-real-time, quasi-global satellite precipitation dataset. *Journal of Hydrometeorology* **21**, 2893–2906 (2020). 34158807 10.1175/jhm-d-20-0177.1 PMC8216223

[20] Sanó, P. *et al*. The passive microwave neural network precipitation retrieval (pnpr) algorithm for the conical scanning global microwave imager (gmi) radiometer. *Remote Sensing* **10**, 1122 (2018).

[21] Amell, A., Hee, L., Pfreundschuh, S., & Eriksson, P. Probabilistic near-real-time retrievals of Rain over Africa using deep learning. *Journal of Geophysical Research: Atmospheres* **130**, e2025JD044595, 10.1029/2025JD044595 (2025).

[22] Kubota, T. *et al*. Advancing global precipitation data products: Recommendations from the international precipitation working group. *Bulletin of the American Meteorological Society* **106**, E564–E570 (2025).

[23] NOAA Climate Prediction Center. Ncep/cpc level-3 merged infrared brightness temperatures (global, 4 km, half-hourly). https://gpm.nasa.gov/data/directory/ncepcpc-level-3-merged-infrared-brightness-temperatures-0 (2025).

[24] Draper, D. W., Newell, D. A., Wentz, F. J., Krimchansky, S. & Skofronick-Jackson, G. M. The global precipitation measurement (gpm) microwave imager (gmi): Instrument overview and early on-orbit performance. *IEEE Journal of Selected Topics in Applied Earth Observations and Remote Sensing* **8**, 3452–3462 (2015).

[25] Goldberg, M. D. & Weng, F. Advanced technology microwave sounder. In Qu, J. J., Gao, W., Kafatos, M., Murphy, R. E. & Salomonson, V. V. (eds.) *Earth Science Satellite Remote Sensing: Vol. 1: Science and Instruments*, 243–253 (Springer, Berlin, Heidelberg, 2006).

[26] Goldberg, M. D., Kilcoyne, H., Cikanek, H. & Mehta, A. Joint polar satellite system: The united states next generation civilian polar-orbiting environmental satellite system. *Journal of Geophysical Research: Atmospheres* **118**, 13–463 (2013).

[27] Berg, W. GPM GMI_R Common Calibrated Brightness Temperatures (Collocated, L1C, 1.5-hour, 13 km, Version 07). https://data.nasa.gov/dataset/gpm-gmi-r-common-calibrated-brightness-temperatures-collocated-l1c-1-5-hours-13-km-v07-gpm-0e459 (2022).

[28] Berg, W. Gpm atms on noaa-20 common calibrated brightness temperatures (l1c, 1.5-h, 17 km, version 07). 10.5067/GPM/ATMS/NOAA20/1C/07 (2022).

[29] Goodman, S. J., Schmit, T. J., Daniels, J. & Redmon, R. J. The GOES-R series: a new generation of geostationary environmental satellites (Elsevier, 2019).

[30] Bessho, K. *et al*. An introduction to himawari-8/9- japan’s new-generation geostationary meteorological satellites. *Journal of the Meteorological Society of Japan. Ser. II* **94**, 151–183 (2016).

[31] Schmetz, J. *et al*. An introduction to meteosat second generation (msg). *Bulletin of the American Meteorological Society* **83**, 977–992 (2002).

[32] Da, C. Preliminary assessment of the advanced himawari imager (ahi) measurement onboard himawari-8 geostationary satellite. *Remote sensing letters* **6**, 637–646 (2015).

[33] Aminou, D. M. A. Msg’s seviri instrument. *ESA Bulletin(0376-4265)* 15–17 (2002).

[34] NOAA Open Data Dissemination Program. Noaa geostationary operational environmental satellites (goes-16, -17, -18 & -19) on aws. https://registry.opendata.aws/noaa-goes/ (2025).

[35] NOAA Open Data Dissemination Program. Jma himawari-8/9 imagery via aws. https://registry.opendata.aws/noaa-himawari/ (2025).

[36] EUMETSAT. Eumetsat seviri dataset - product eo:eum:dat:0962. https://data.eumetsat.int/product/EO:EUM:DAT:0962 (2025).

[37] Hersbach, H. *et al*. The era5 global reanalysis. *Quarterly journal of the royal meteorological society* **146**, 1999–2049 (2020).

[38] Passive Microwave Algorithm Team Facility. Gpm gprof algorithm theoretical basis document (atbd), version 7. Tech. Rep., NASA Global Precipitation Measurement (GPM) Mission (2022).

[39] National Centers for Environmental Information. Daily automated snow and ice cover maps for the northern hemisphere and southern hemisphere. https://www.ncei.noaa.gov/access/metadata/landing-page/bin/iso?id=gov.noaa.ncdc:C01697 Accessed: Aug. 22, 2025 (2025).

[40] Hastings, D. A. & Dunbar, P. K. Global land one-kilometer base elevation (globe). https://repository.library.noaa.gov/view/noaa/13424 Accessed: Aug. 14, 2025 (1999).

[41] Smith, T. M. *et al*. Multi-radar multi-sensor (mrms) severe weather and aviation products: Initial operating capabilities. *Bulletin of the American Meteorological Society* **97**, 1617–1630 (2016).

[42] Kwon, S., Jung, S.-H. & Lee, G. Inter-comparison of radar rainfall rate using constant altitude plan position indicator and hybrid surface rainfall maps. *Journal of Hydrology* **531**, 234–247 (2015).

[43] Ryu, S., Song, J. J. & Lee, G. Radar-rain gauge merging for high-spatiotemporal-resolution rainfall estimation using radial basis function interpolation. *Remote Sensing* **17**, 530 (2025).

[44] Sohn, B. J., Ryu, G.-H., Song, H.-J. & Ou, M.-L. Characteristic features of warm-type rain producing heavy rainfall over the korean peninsula inferred from trmm measurements. *Monthly Weather Review* **141**, 3873–3888 (2013).

[45] Fuchsberger, J., Kirchengast, G. & Kabas, T. Wegenernet high-resolution weather and climate data from 2007 to 2020. *Earth System Science Data* **13**, 1307–1334 (2021).

[46] Tan, M. & Le, Q. Efficientnetv2: Smaller models and faster training. In *International conference on machine learning*, 10096–10106 (PMLR, 2021).

[47] Kidd, C. & Huffman, G. Global precipitation measurement. *Meteorological Applications* **18**, 334–353 (2011).

[48] Pfreundschuh, S. *et al*. A neural network approach to estimating a posteriori distributions of Bayesian retrieval problems. *Atmospheric Measurement Techniques* **11**, 4627–4643 (2018).

[49] ipwgml/satrain: Satrain v1.0 10.5281/zenodo.17743454 (2025).

[50] Pfreundschuh, S. Satellite precipitation estimation evaluation data (speed) package. GitHub repository accessed 2025-09-06 https://github.com/ipwgml/speed (2025).

